# Translational medicine for acute lung injury

**DOI:** 10.1186/s12967-023-04828-7

**Published:** 2024-01-05

**Authors:** Jianguo Zhang, Yumeng Guo, Michael Mak, Zhimin Tao

**Affiliations:** 1 Department of Emergency Medicine, The Affiliated Hospital, Jiangsu University, Zhenjiang, 212001 Jiangsu China; 2 Jiangsu Key Laboratory of Medical Science and Laboratory Medicine, Department of Laboratory Medicine, School of Medicine, Jiangsu University, Zhenjiang, 212013 Jiangsu China; 3 Department of Biomedical Engineering, School of Engineering and Applied Science, Yale University, New Haven, 06520 USA; 4 Zhenjiang Key Laboratory of High Technology Research on Exosomes Foundation and Transformation Application, School of Medicine, Jiangsu University, Zhenjiang, 212013 Jiangsu China

**Keywords:** Acute lung injury, Acute respiratory distress syndrome, Animal model, Nanomedicine, Drug delivery, Therapy

## Abstract

Acute lung injury (ALI) is a complex disease with numerous causes. This review begins with a discussion of disease development from direct or indirect pulmonary insults, as well as varied pathogenesis. The heterogeneous nature of ALI is then elaborated upon, including its epidemiology, clinical manifestations, potential biomarkers, and genetic contributions. Although no medication is currently approved for this devastating illness, supportive care and pharmacological intervention for ALI treatment are summarized, followed by an assessment of the pathophysiological gap between human ALI and animal models. Lastly, current research progress on advanced nanomedicines for ALI therapeutics in preclinical and clinical settings is reviewed, demonstrating new opportunities towards developing an effective treatment for ALI.

## Background

**Fig. 1:**
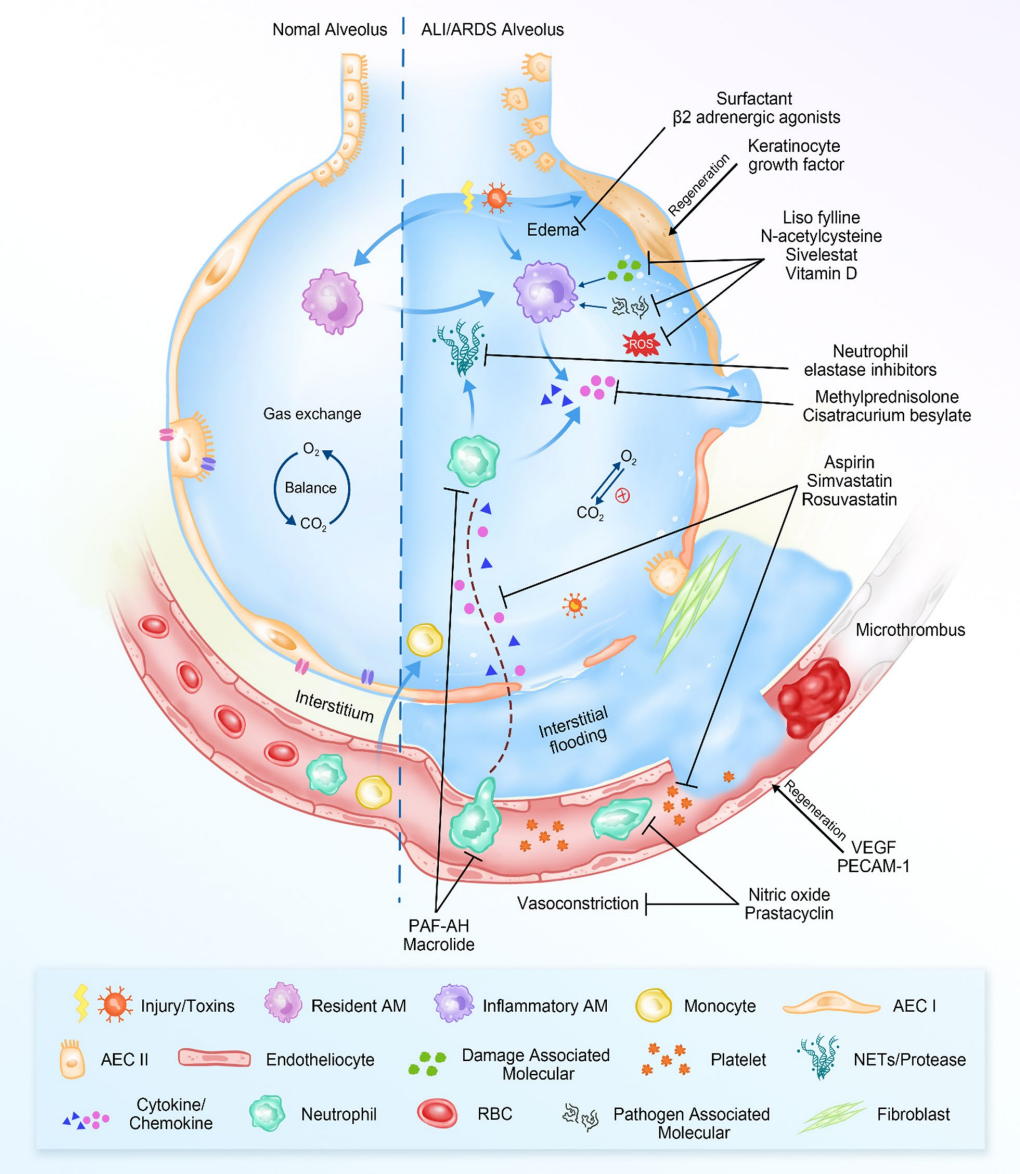
Graphics indicating the normal alveolus and ALI/ARDS alveolus. The right alveolus is a schematic representation of the main pathophysiological features of ALI/ARDS. Each therapeutic agent acts through a specific mechanism as indicated and these machineries in detail were reviewed elsewhere [181, 182]. PECAM-1 = platelet-endothelial cell adhesion molecule-1, VEGF = vascular endothelial growth factor, PAF-AH = platelet-activating factor acetyl hydrolase

Human lung structure evolves to facilitate O_2_-CO_2_ exchange in the alveolar-capillary unit, where microvascular endothelium, interstitium, and alveolar epithelium form an intact alveolar-capillary membrane (ACM) as a selective barrier to prevent protein-rich fluid diffusion and non-gaseous solute passage (Fig. 1) [1]. On one side of ACM is a continuum of type I and II alveolar epithelial cells (AEC) that interacts with resident alveolar macrophages to modulate immune responses or counteract inflammations primarily from the lower respiratory tract [2]. On the other side of ACM, a lining of microvascular endothelium along capillaries regulates vessel permeability and maintains tissue hemostasis by secreting a variety of active enzymes [3].

Acute lung injury (ALI) is characterized by a sudden decrease in lung gas exchange function caused by extrapulmonary (indirect) or pulmonary (direct) insults such as lung infection, trauma, and other non-cardiogenic pathogenic factors [4]. These injuries compromise ACM integrity, resulting in signaling of inflammatory cytokines and the release of immune cells (e.g., platelets, leukocytes) and proteins into the alveolar airspace (Fig. 1) [5]. Consequently, gas exchange is obstructed, and edema develops, causing diffuse alveolar damage (DAD) and the formation of a hyaline membrane along the alveolar walls, which is visible radiologically as patchy ground-glass patterns [6]. Regardless of whether the initial challenges are pulmonary or extrapulmonary, the outcomes share common pathological features, such as a compromised alveolar-capillary barrier, interstitial or interalveolar edema formation, impaired/damaged gas exchange capacity, and respiratory failure, despite differences in lung elastance, chest imaging, respiratory mechanics, and treatment strategies [7].

After the initial lung injury, a self-repair machinery may be activated. AEC II pneumocytes can proliferate and differentiate into AEC I pneumocytes, allowing edema fluids to drain into the interstitium and calling macrophages to purge cell debris [2, 8]. Through this, the ACM integrity may be restored to some extent, improving oxygenation; or it may be unable to remove alveolar fluid, leading to hypoxemia and hypercapnic acidosis, and ALI progresses to acute respiratory distress syndrome (ARDS) [9].

Concurrently, a fibrotic phase may occur when cystic changes in mesenchymal cells cause collagen accumulation, forming intra-alveolar, interstitial, and/or capillary fibrosis [10]. Continuous inflammation and progressive fibrosis become important predictors of a worsened ALI/ARDS outcome, which is typically associated with increased mortality [10]. Regardless of whether DAD is a histopathological hallmark of ARDS, it only occurs in about one-half of ARDS patients, but the presence of DAD could double the mortality risk [11]. Other ARDS settings where DAD is absent can also show distinct neutrophil permeation into the alveolar airspace rather than hyaline membrane formation [12]. When compared to extrapulmonary causes of ARDS (e.g., peritoneal sepsis), direct pulmonary causes of ARDS (e.g., pneumonia) showed a greater proclivity for alveolar damage and interstitial edema, leading to significantly reduced oxygenation, though the outcome could be similar [13].

The clinical definition of ARDS was first established in 1994 and was updated in 2011 when the Berlin definition provided the diagnostic criteria for ARDS, primarily based on patients’ oxygenation index (PaO_2_/FiO_2_) [14, 15]. ARDS is classified into three categories: mild (200 mmHg < PaO_2_/FiO_2_ ≤ 300 mmHg), moderate (100 mmHg < PaO_2_/FiO_2_ ≤ 200 mmHg), and severe (PaO_2_/FiO_2_ ≤ 100 mmHg), with mortality rates of 27%, 32%, and 45%, respectively, and a median duration of 5, 7, 9 days using mechanical ventilation in survivors [15]. Since then, various recommendations have been made for ARDS patient management [16, 17]. For example, for ARDS patients on mechanical ventilation, a low tidal volume of < 6 mL/kg ideal body weight and an airway pressure of < 30 cmH_2_O were recommended [17].

According to a large study conducted years ago that included 459 intensive care units (ICUs) from more than 50 countries, general ARDS mortality in ICU patients was estimated to be 35.3% [18]. Many factors can influence mortality, including the primary diagnosis, disease cause, and medical condition. During epidemics of infectious respiratory illness, ALI/ARDS mortality after ICU admission can be much higher. For example, 67–85% and 52.2% mortality were reported for ICU patients who died of ARDS during the early period of the coronavirus disease 2019 (COVID-19) pandemic and the 2002–2003 severe acute respiratory syndrome (SARS) regional outbreak, respectively [19–21].

Currently, there are no clinically approved drugs that can effectively reduce mortality of patients with ARDS [22]. Experimental drugs aimed at different biological targets for ALI/ARDS therapy are in various stages of clinical trials (Table 1), but no improved therapeutic outcome has been confirmed [23]. For example, low-dose nitric oxide inhalation or intratracheal surfactant instillation can effectively improve oxygenation in patients with ALI, but it does not significantly reduce mortality [24, 25]. Instead, the most effective treatment is a medical intervention aimed at reducing pulmonary or/and systemic inflammation, in conjunction with supportive therapy based on mechanical ventilation [26]. Furthermore, prone positioning for patients with moderate and severe ARDS on mechanical ventilation may improve survival rates, possibly due to more effective tidal volume allocation [27]. For the treatment of patients with severe ARDS, the extracorporeal membrane oxygenation (ECMO) technique improves blood oxygenation, despite its potential to increase the bleeding risk of patients with ARDS [28].

**Table 1:** A list of investigational therapeutic agents for ALI/ARDS at various stages of clinical trials obtained via www.ClinicalTrials.gov as of December 23, 2023

Intervention	Proposed mechanism	Primary outcome measures	Phase; NCT#
Almitrine	Oxygenation improvement by increasing hypoxic pulmonary vasoconstriction	Increase in PaO_2_/FiO_2_ ratio	Not applicable; NCT05216575
Anti-interleukin drugs (e.g., Tocilizumab)	Inhibiting secretion of inflammatory factors to alleviate lung damage	Time to clinical improvement	Phase 3; NCT04330638
Budesonide and formoterol	FDA-approved treatment for asthma and chronic obstructive pulmonary disease	Oxygen saturation to fraction of inspired oxygen concentration (SpO_2_/FiO_2_) ratio	Phase 2; NCT01783821
Cysteamine-pantetheine disulfide	Antiviral, anti-infectious, antioxidant and anti-CRS (cytokine release syndrome)	28-day hospitalization or mortality	Phase 2; NCT05212662
Dexamethasone	Immunomodulatory properties to decrease the patients’ mortality	Development of moderate-severe ARDS	Phase 3; NCT04836780
Granulocyte macrophage-colony stimulating factor	Improvement of host defense and lung barrier restoration	Mononuclear phagocyte activation/polarization in BALF	Phase 2; NCT02595060
Nitric oxide	Previous beneficial effects on SARS-CoV infected epithelial cells	Reduction in requirement of intubation and mechanical ventilation	Phase 2; NCT04305457
Pirfenidone	Reducing lung fiber proliferation and idiopathic pulmonary fibrosis in ARDS	The number of ventilator-free days at day 28	Phase 3; NCT05075161
Polyvalent immunoglobulin	Attenuating the inflammatory reaction and strengthening the antiviral response	Ventilator-free days	Phase 3; NCT04350580
Siverestat sodium	Inhibition of neutrophil elastase and blockade of inflammatory cascade	Oxygenation improvement rates and ventilator-free days	Phase 4; NCT04909697
Triiodothyronine (T3)	Intervening in the production of pulmonary fibrosis and relieving inflammation and oxidative stress	The extravascular lung water index	Phase 2; NCT04725110
Tissue plasminogen activator (Alteplase)	Targeting coagulation and fibrinolytic systems to ameliorate ARDS	Increase of PaO_2_/FiO_2_ from pre-to-post intervention	Phase 2; NCT04357730
Ulinastatin	Inhibiting a variety of inflammatory proteases	Reduction of ARDS incidence	Not applicable; NCT03089957
Umbilical cord derived exosomes and stem cells	Alleviating the pulmonary distress in COVID-19 related ARDS	Assessment of treatment-emergent adverse events and treatment efficacy	Phase 1; NCT05387278
Vitamin C	Mitigating the dysregulated transformation from infection into sepsis	Number of participants deceased or with persistent organ dysfunction	Phase 3; NCT04404387
Zilucoplan^®^	A complement C5 inhibitor that promotes lung repair mechanism	Increase in PaO_2_/FiO_2_ ratio	Phase 2; NCT04382755

(Condition/disease =acute lung injury/acute respiratory distress syndrome; Study Status = not yet recruiting/recruiting/active not recruiting/completed)

### Translational ALI models

ALI is a complex illness with multiple paths of pathogenesis and various responses to therapeutics and disease outcomes [29]. The currently accepted definition of ALI was articulated in the 1992 American-European Consensus Conference, organized by the American Thoracic Society and the European Society of Intensive Care Medicine, and published in 1994 [30]. The recommended ALI criteria are acute onset, a low oxygenation index (PaO_2_/FiO_2_) ≤ 300 mmHg, bilateral infiltrates in the chest radiograph, and a pulmonary artery wedge pressure of ≤ 18 mmHg or no clinical evidence of left atrial hypertension, whereas ARDS could be at the severe end of this pathological process [14].

In basic research on human diseases, animal studies are always an important link between bench work and clinical trials. However, unlike many other disease models, translational ALI research is a particularly difficult task, for several reasons. First, establishing the above-mentioned ALI criteria are impractical in animal models due to incompatibility in ALI-related anatomical structures and physiological functions between laboratory animals and humans [31]. For example, animal lung size and pulmonary immunity may limit their meaning in humans. Second, despite enormous efforts to find biomarkers for ALI diagnosis and prognosis in recent decades, clinically and experimentally valid measures with high specificity and sensitivity remain lacking [32]. Third, because ALI is a heterogeneous disorder, it can be classified into phenotypes with distinct clinical features and outcomes [33]. For example, according to diverse injurious natures, ALIs are commonly classified as either direct (intrapulmonary) or indirect (extrapulmonary), with very few similarities in pharmacological or therapeutical approaches. Thus, insults used to build animal models and the resulting responses vary greatly in experimental ALI studies, making them difficult to interpret and sometimes contentious. For these reasons, it is known that no animal models can fully represent the biological characteristics of ALI [34].

Next, we compare various small animal models to characterize the pros and cons of each specified ALI model (Table 2) and highlight the key determinants in appropriately building such animal models. Following are direct and indirect ALI models based on various etiologies, including typical protocols of experimental procedures and the associated pathogenetic mechanisms.

**Table 2:** Comparison of advantages and shortcomings of common ALI models with different etiologies

Method	Advantage	Shortcoming	Pathogenesis
Direct ALI models			
Endotoxin challenge	Simple usage	The quality of reagents used may vary significantly	Direct exposure of bioactive microbial units to lungs
Bacterial infection	High relevance to real-world scenario of ALI	Experiments require biosafety concern and attention	Direct impact of endotoxin on lung tissues
Acidic instillation	Direct and confined damage to airways	This does not resemble gastric acid aspiration	Injury of alveolar epithelium caused by acidic insults
Bleomycin exposure	Facile experimentation	Toxicity could be various and more than pulmonary	Induction of pulmonary fibrosis
Oleic acid induction	Facile experimentation with high reproducibility	Delivery of insoluble oleic acid to airways is ineffective	Formation of pulmonary microvascular embolism
Ventilation induction	Close relevance to clinical practice	Injurious patterns are significantly different between animals and human	Severe mechanical overdistention of lung structure
Pulmonary contusion	High relevance to physical trauma	Low repeatability	Formation of thrombi in pulmonary capillaries
Indirect ALI models			
IR-induced	Consistency with pathogenic reperfusion in clinical settings	Complex operation with poor reproducibility	Increased alveolar permeability due to excessive pro-inflammatory cytokines produced by immunity
Transfusion-caused	Clinically related with facile experimentation	Low repeatability and high dependency on blood products used	Formation of neutrophil sequestration and pulmonary edema
Acute pancreatitis-associated	High clinical relevancy	Complicated experimentation with unpredictable outcome	Endothelial damage and vascular permeability in lungs
CLP/CASP	High occurrence in clinical practice	Surgical operation requires sufficient skillfulness	Secondary lung impairment following intra-abdominal sepsis

### Direct ALI models

In experimental settings, direct ALI models are activated by intrapulmonary factors such as endotoxin challenge, microbial infection, chemical induction (e.g., oleic acid, hydrochloric acid), ventilator intubation, and physical/radiative impairment (e.g., contusion, radiation), etc. The endotoxin challenge, which was originally designed to mimic microbial infection through inhalation and the clinical development of human ARDS, is the most popular among them [35, 36]. The bacterial membrane component of lipopolysaccharide (LPS) is typically administered via intratracheal instillation or aerosolization, to cause ALI in rodent models, resulting in increased microvascular permeability, elevated neutrophil migration into the airspace, and profound pulmonary inflammation [35, 36]. In contrast, intraperitoneal or intravenous administration of LPS to mice resulted in milder ALI with more transient inflammation, more resembling septic infection than direct lung injury [37]. It is worth noting that in LPS-induced ALI models, the impurity of the LPS reagent used varies, which may initiate the ALI cascade via different signaling pathways [38], and the ALI events triggered are dependent on oxidative responses but independent of complement activation [39, 40]. Similarly, intratracheal administration of ambient particulate matter, such as diesel exhaust particles or cigarette smoke, was found to stimulate ALI in preclinical studies by activating Toll-like receptors and releasing proinflammatory cytokines [41].

Given that pneumonia is the most common cause of ARDS, with sepsis as the primary linkage [42], microbial infection has been widely used to develop pneumonia-associated ALI models. A variety of respiratory pathogens including live bacteria and viruses have been introduced into animal lungs via methods such as aerosolization, transtracheal injection, intranasal inoculation, etc. [43]. Because the infection is always the result of an interaction between the invading substance and the host immunity, at least three factors should be prudently considered before we can translate animal data into meaningful results that fit the human clinical scenario. First, the same pathogen used in animal studies has very different clones and serotypes, resulting in a wide range of pulmonary injury potentials [44]. Second, for certain species-specific pathogens, animal models may not be infected at all, necessitating the use of a transgenic, transplanted, and/or humanized model, or they may produce varying injurious responses depending on the strain, sex, and/or age, etc. [45, 46]. For example, intranasal instillation of a highly infective strain of *Rodentibacter pneumotropicus* in C57BL/6 and BALB/c mice resulted in distinct antibody productions and lung pathologies, resulting in different mortalities [47]. Furthermore, after receiving SARS-CoV-2 via intratracheal instillation, C57BL/6 mice with human angiotensin-converting enzyme 2 (hACE2) expression exhibited pathological changes in the lungs similar to human ALI/ARDS following COVID-19 infection, including bilateral congestion, pulmonary edema, and hyaline membrane formation, which had previously not been observed in other murine models [48]. Third, the interactions between the infectious pathogen and the animal host are influenced by an infinite number of genetic and environmental factors, complicating the experimental results. For example, bacterial co-infection is very common in virally infected ALI patients who require intensive care, and this may not be well replicated in animal studies. Furthermore, patient comorbidity is a known risk factor for poor ALI/ARDS prognosis, but how it can be tested in animal models remains elusive [31, 45].

To imitate the aspiration of gastric contents into the lower respiratory tract that leads to ALI/ARDS, hydrochloric acid (HCl) has been instilled intratracheally or orotracheally in animal models, directly targeting the alveolar epithelium [49]. Consequently, chemical insults have a pathogenic effect 4–6 h post-instillation, which is orchestrated by alveolar-capillary barrier dysfunction, the intra-alveolar elevation of proinflammatory cytokines (tumor necrosis factor-α in mice), and increase of neutrophil infiltration (mediated by interleukin-8 in rabbits), and collapse of alveolar fluid transport [50, 51]. However, caution should be exercised when using HCl-induced ALI animal models. First, small changes in acidic concentrations can significantly alter the outcome of lung damage, and the dosage windows between no injury and fatal injury are quite narrow [49]. Second, human aspirates contain a wide range of chemical fluids, biological secretions, and solid particles, including stomach acids, blood, bacteria, and food particles, which may contribute to a distinctive ALI pathogenesis from HCl-induced aspiration [52]. Third, after HCl instillation, animals are usually ventilated to improve survival rates, and acidic pH (if moderate) can be rapidly neutralized in the lungs without severe injury [53]. Thus, a two-hit model (e.g., HCl followed by LPS) may be preferable for developing an extended injury model and studying long-term ALI sequela and therapeutic interventions [54].

Other common chemicals to induce direct ALI models are bleomycin and oleic acid. Bleomycin is an antibiotic and anticancer drug with a major side effect of pulmonary toxicity, which manifests as lung fibrosis in a significant proportion of cancer patients [55]. As a result, this drug has been used to induce ALI in animal models via intravenous, intraperitoneal, or intratracheal administration, resulting in DNA damage, free radical production, oxidative stress, inflammatory response, and pulmonary fibrosis [56]. Additionally, in vitro 3D tissue models utilizing human cells, including from normal and diseased donors, have potential for capturing dynamic and patient specific events relevant to disease progression and provide a scalable platform for screening and development of novel therapeutics [57–59]. It should be noted that dosage, route of administration, and animal status (e.g., age, gender, strain) may all affect susceptibility to lung injuries and produce different injurious sites [60]. Furthermore, oleic acid is an 18-carbon unsaturated fatty acid that is intravenously administered in large animal models (e.g., sheep, dog) to mimic lipid embolism in the pulmonary circulation, which predisposes to non-septic ARDS [61]. The oleic acid-induced ALI model is a classic one that is highly reproducible across animal species, causing direct and immediate (within minutes) damage to capillary endothelium via necrotic cell death, followed by interstitial edema, epithelial injury, neutrophil penetration, and diffuse hemorrhage [62]. Nonetheless, because oleic acid is not water-soluble, it must be dissolved in water-miscible organic solvents (e.g., ethanol) or mixed with blood or saline before administration in vivo. This preparation may produce different hemodynamic responses and pulmonary permeabilities depending on the bioavailable oleic acids, altering the pathological outcomes [61]. Furthermore, because oleic acid-induced ALI does not involve excessive inflammation, it is unsuitable for most sepsis-caused ALI/ARDS studies [31, 63].

Clinically, mechanical ventilation is a standard therapy to improve gas exchange in ALI/ARDS patients, but improper use can exacerbate critical condition by causing ventilator-induced lung injury (VILI) [64]. The pathogenic consequences of VILI include increased permeability of the alveolar-capillary barrier, resulting in pulmonary edema, and mechanical overdistention of the lung structure, resulting in inflammation [65]. Although the causes of VILI are unknown, the causative factors point to inappropriate plateau airway pressures, tidal volumes, and positive end-expiratory pressure (PEEP) [66]. In the case of moderate to severe ARDS, the official recommendations include low tidal volumes of 4–8 mL/kg predicted body weight, low plateau pressure less than 30 cm H_2_O, and conditionally high PEEP (normally greater than 5 cm H_2_O) [16, 17]. When it comes to animal models of VILI, any variable influencing the interaction between ventilators and animal subjects could alter the injuring patterns such as mechanical force, tidal volume, airway pressure, and animal conditions such as species or strains, body temperature, respiratory rate, anesthetic choice, etc. [67]. In contrast to invasive mechanical ventilator intubation in VILI, hyperoxia-induced ALI (or hyperoxic acute lung injury, HALI) results from non-invasive lengthened breathing of high FiO_2_ (fraction of inspired oxygen), causing pulmonary O_2_ toxicity with serious consequences on gas exchange and respiratory mechanics [68]. The reactive oxygen species (ROS) produced further initiates cell death, activates the inflammatory cascade, and exacerbates alveolar damage, potentiating lung injuries that are highly dependent on oxygen concentration and exposure duration [69]. Because supportive care with mechanical ventilation and high concentrations of oxygen is common in ICU settings, HALI/VILI combinations have frequently been brought to laboratory animal research in search of a better remedy [70].

Other direct injuries used to induce preclinical ALI models include repeated saline lavage, pulmonary contusion, and radiation therapy. In fact, repeated lavage with warm isotonic saline to deplete pulmonary surfactants undermines alveolar stability and lung compliance, resulting in epithelial damage, which is reversed in human ALI pathogenesis [63]. Thus, this method is better suited for use in a two-hit animal model after the first triggering factor of ALI is applied. Furthermore, lung contusion (invasive) or radiation (non-invasive) induced ALI models can be developed to better understand the acute inflammatory and injurious responses, with lung fibrosis as the result [56]. Although these models are simple and reproducible, their relevance to human ALI/ARDS research is limited, and they rarely suggest meaningful therapeutic strategies [71].

### Indirect ALI models

As opposed to direct pulmonary stimuli, indirect methods to induce ALI in animal models contribute to a smaller portion of preclinical ALI research with the extreme heterogeneity of ALI pathomechanisms [72]. Ischemia–reperfusion (IR), transfusion, and extrapulmonary sepsis are the most common factors triggering ALI indirectly.

Among these, IR-induced ALI is one of the most serious postoperative complications following cardiothoracic or pulmonary surgeries, such as lung transplantation and cardiopulmonary bypass, with a high mortality rate [73]. Ischemia-induced ROS is a key player in IR-induced lung damage, and during reperfusion, it activates lymphocytes, neutrophils, and platelets, causing them to release proinflammatory cytokines and prothrombotic factors, perpetuating vascular damage and permeability [74]. In preclinical studies, three major steps determine the success of experimental modeling: (1) large animal species (e.g., sheep, pig, dog) are typically chosen for their ease of operation and high tolerance; (2) during an ischemic phase, the extent of ischemic bed (including the pulmonary artery, bronchial circulation, venous return, and alveolar ventilation) and its duration time must be well controlled; (3) a two-phase reperfusion should be closely monitored because the first-half acute phase (~ 30 min after reperfusion) is neutrophil-independent and the second-half delayed phase (> 4 h in the following) shows more noticeable pathological changes in the lung [75]. As a result, alveolar damages are observed, such as neutrophil sequestration, edema formation and pulmonary hemorrhage [75].

Clinically, within 6 h or extended to 6–72 h after blood or blood component administration, transfusion-related ALI (TRALI) has become the leading cause of transfusion-related fatality, transforming a life-saving benefit into a life-threatening risk [76]. Among all patients receiving a blood transfusion, TRALI occurs in 0.08–15%, with ICU patients having a 50–100 times higher occurrence than general inpatients, which is consistent with the fact that 50–70% of critical patients require a blood transfusion during their ICU stays [76]. Although TRALI is complicated and multifactorial, its etiology can be posited as a two-hit model, where the first hit is associated with the patient/recipient condition, such as inflammation and sepsis, and the second hit is associated with blood transfusion, including blood product quality and donor-recipient histocompatibility [77]. In animal models, TRALI is typically induced by neutrophil priming with intratracheal or intravenous LPS before intravenous infusion of anti-neutrophil/human leukocyte antigen (HLA)/major histocompatibility complex antibodies or aged blood products, resulting in neutrophil sequestration and pulmonary edema [78]. However, TRALI is not reproducible in animal models, and the effective TRALI-triggering dosages of alloantibodies or other second hit reagents are unknown [78].

Acute pancreatitis is an inflammatory condition that, in severe cases, can result in multiple organ failure, ALI/ARDS, and death [79]. The pathophysiology of acute pancreatitis-associated lung injury in humans begins with an impaired pancreas and the subsequent release of digestive enzymes into the systemic circulation, such as phospholipase A_2_, elastase, and trypsin, which further induces endothelial breakage and vascular permeability in the lungs [80]. Animal models of pancreatitis have been well established for studying the resulting ALI; however, the clinical relevance of such models is usually low, and in most scenarios, no ALI can be observed [79]. Similarly, cecal ligation and puncture (CLP) and colon ascendens stent peritonitis (CASP) models have been developed to study ALI secondary to peritonitis, emulating the intra-abdominal infections in humans that can lead to sepsis and ALI/ARDS [81]. The murine models CLP and CASP share a basic concept in that perforation is introduced by either puncturing the cecum or inserting a stent into the ascending colon, allowing fecal materials to leak into the intraperitoneal cavity and cause polymicrobial sepsis [82]. However, no significant ALI/ARDS is frequently observed in those models because pulmonary complications are rarely the cause of animal death [83]. Instead, they are better suited for sepsis study than ALI research.

### Heterogeneity of human ALI/ARDS

Large epidemiology studies on ALI patients found that clinical risk factors of intrapulmonary origins, such as pneumonia, lung contusion, and aspiration, etc., account for the majority of ALI causes (up to ~ 80%), while those of extrapulmonary origin, such as non-pulmonary sepsis and non-cardiogenic shock, account for a small portion [84]. Furthermore, a higher incidence of ALI with a direct pulmonary source may be associated with a higher frequency of ARDS and a higher mortality rate [18, 84], possibly due to poorer respiratory mechanics and lung elastance when compared to indirect insults [85]. Nevertheless, controversial reports have emerged claiming that both direct and indirect ALI/ARDS caused comparable overall mortality, although the severity of illness may differ [7, 86]. Yet the long-term pulmonary sequelae after 6 month clinical management of patients with direct or indirect ARDS etiology indicated no statistical differences [87].

Accordingly, distinguishable features in chest radiography and computed tomography (CT) images were discovered between patients with ARDS caused by pulmonary and extrapulmonary injury, where alveolar consolidation and ground-glass opacification predominated separately [88]. Those with pulmonary origins had more extensive nondependent consolidation and parenchymal cysts, but less extensive dependent intense parenchymal opacification [89]. Simultaneously, ARDS of pulmonary origin had higher lung elastance but lower intra-abdominal pressure and chest wall stiffness [90].

The search for reliable ALI/ARDS biomarkers has been active in the last decade [91]. Novel techniques including proteomics analyses based on advanced liquid chromatography and mass spectroscopy have been used to compare a variety of biological fluids (e.g., plasma serum, bronchoalveolar lavage fluid or BALF) from patients with ALI/ARDS to those from healthy controls, identifying key molecules, major pathways, and primary drug targets in this complex disease [92]. According to one pilot study, differentially expressed proteins were enriched in the acute phase of ALI, regardless of direct or indirect insults, and the majority were involved in the biological processes of lipid transport and complement activation [93]. Furthermore, a panel of inflammation-associated microRNAs (miRNAs) was found to be upregulated in patients with ALI/ARDS, with miR-155/-887-3p and miR-27a/126 being linked to sepsis- and pneumonia-related ALI, respectively [94].

Pathological pieces of evidence confirmed that patients with direct ARDS had localized damage to the lung epithelium, whereas patients with indirect ARDS had a systemic endothelial injury caused by diffuse vascular inflammation, where surfactant protein D and angiopoietin-2 were identified as specific plasma biomarkers of direct and indirect ARDS, respectively, both of which were prognostic of mortality [95]. Higher levels of inflammatory cytokines and neutrophil activation were consistently associated with ARDS of pulmonary origin, resulting in increased pulmonary permeability [7]. In the meantime, endothelial biomarkers like von Willebrand factor and vascular cell adhesion molecule were closely linked to indirect ALI [96]. Unfortunately, no single biomarker has been validated for accurate ALI/ARDS diagnosis or prognosis.

Genetic determinants, as opposed to protein biomarkers transiently expressed during ALI pathogenesis, aid in determining an individual’s susceptibility, severity, and mortality from ALI/ARDS [97]. Genome-wide association study (GWAS) revealed that *PPF1A1* and *SELPLG* were identified as ALI/ARDS risk genes in European American and African American populations, respectively [98, 99]. The expression-based GWAS approach identified the *CLEC4E* (C-type lectin domain family 4 member E) gene, which codes for the pattern recognition receptor of the innate immune system, as a novel biomarker of ALI [100], possibly triggered by direct stimuli. Gene polymorphism has also been linked to ALI pathogenesis, such as insertion/deletion in the angiotensin-converting enzyme gene [101], and variant alleles of Pre-B-cell colony-enhancing factor (PBEF)[102], interleukin 18 (IL-18) [103], and mannose-binding lectin-2 (MBL-2) [104]. Furthermore, single nucleotide polymorphisms in the *POPDC3* (Popeye domain-containing protein family) and fatty acid amide hydrolase genes contributed to direct and indirect ALI/ARDS development, respectively [105].

Diverse interactions between genes and host/environment result in a variety of non-genetic risk factors for ALI/ARDS incidences across age, gender, and ethnic groups [106]. Based on the inconsistent cohort sizes and ALI causes, reports on the effect of gender on ALI/ARDS susceptibility, severity, and mortality remain contradictory, although proinflammatory sex hormones play an essential role in ALI pathogenesis [107]. Non-white ethnicities and older age were linked to an increased ALI incidence and mortality [108]. While chronic alcoholism caused ethanol-mediated glutathione deficiency in human lungs, passive or active smoking increased plasma cotinine levels, both of which increased susceptibility to ALI [109]. Furthermore, pre-existing diseases, particularly those that cause liver failure and immune incompetence, were linked to an increased incidence and death rate from ALI/ARDS [110]. However, little is known about how and to what extent these risk factors contribute to the differences between pulmonary and extrapulmonary ALI.

### Treatment for ALI/ARDS

The treatment of ALI/ARDS patients primarily consists of non-pharmacological aeration and pharmacological intervention. Mechanical ventilation, positive end-expiratory pressure (PEEP), recruitment maneuver, and prone positioning are among the aeration strategies used to provide breathing support and improve patients’ oxygenation [111]. For all ventilated ARDS patients with mixed injurious causes, low tidal volume ventilation (normalized to predicted body weight) has been recommended to reduce ICU mortality [112]. ARDS patients with pulmonary origin had a lower PaO_2_/FiO_2_ ratio and intra-abdominal pressure than those with extrapulmonary origin, but had higher lung recruitability in the early phase [13]. Virtually, alveolar recruitments were nearly comparable in pulmonary and extrapulmonary ARDS when induced by different PEEP levels (10 or 14 cm H_2_O) [113]. Furthermore, increasing PEEP (0-15 cm H_2_O) in pulmonary ARDS patients resulted in increased elastances of both the lung and respiratory system, whereas the opposite was true in extrapulmonary ARDS [114].

To avoid shear stress, as a result of mechanical ventilation and a major cause of VILI, the open lung strategy has been used, which involves using a recruitment maneuver to homogenously re-aerate the deflated alveoli while tuning a proper PEEP level to maintain alveolar stability [115]. Sustained inflation and (extended) sigh are common recruitment tactics [116]. By maintaining positive airway pressure for 30 s, the PaO_2_/FiO_2_ ratio was improved in both ARDS subtypes, followed by high PEEP (~ 16 cm H_2_O), which only improved the elastance of the respiratory system in extrapulmonary ARDS patients [117]. Alternatively, keeping three consecutive sighs per minute at a plateau pressure of 45 cm H_2_O, patients with ARDS showed significantly better oxygenation and lower lung elastance than those with no sigh periods, indicating that this ventilatory treatment has greater effectiveness and recruitment potential in ARDS of extrapulmonary origin [118]. In rat models, recruitment maneuvers were found to be more effective in improving oxygenation and lung mechanics in patients with extrapulmonary ALI [119]. Despite the fact that all of these findings demonstrated a reduction in lung structure damage caused by ventilation, recruitment maneuvers are only recommended when a life-saving decision must be made [116]. Simultaneously, in comparison to traditional lung protective ventilation using low tidal volume, a combination of low tidal volume, recruitment maneuver, and high PEEP did not result in a reduction in ALI mortality, despite significantly improving their refractory hypoxemia [120].

Prone positioning has been used to improve the oxygenation of ARDS patients for decades, although it does not reduce mortality [121]. Whether the responses to prone position differ between pulmonary and extrapulmonary ARDS is still unclear. With nitric oxide inhalation, the prone position increased the oxygenation more significantly than the supine position, regardless of whether the cause of ARDS was direct or indirect [122]. In contrast, within 3 days of ARDS onset, the prone position enabled patients of extrapulmonary origin to improve their oxygenation and respiratory system compliance more quickly and significantly (e.g., atelectasis reversal, consolidation elimination) than those of pulmonary origin [123]. Collectively, prone positioning is a difficult respiratory care challenge because forcing an intubated patient into an unnatural posture necessitates professional intervention and may result in individual consequences [124].

Since its inception, venovenous extracorporeal membrane oxygenation (VV-ECMO) has evolved into a cutting-edge life-saving technology for patients suffering from ARDS when their blood is circulated via cardiopulmonary bypass for artificial CO_2_-O_2_ exchange [125]. When compared to lung protective ventilation using low tidal volumes and airway pressures, prone positioning or ECMO was individually associated with lower 28-day mortality in patients with ARDS [126]. Furthermore, ECMO with extended prone positioning hours improved both oxygenation and respiratory system compliance without causing significant harm [127], particularly in SARS-CoV-2 infection-induced ARDS [128]. Recently, patients with severe ARDS had significantly lower 28-, 60-, and 90-day hospital mortality rates in the subtype of direct injury, despite having higher lung injury scores and being on ECMO longer [129]. Nonetheless, ECMO use in the early wave of the COVID-19 pandemic has been linked to improved morale in hospitalized COVID-19 patients with ARDS, emphasizing the importance of experienced ECMO initiation and evolving decision making [130].

Undoubtedly, oxygen therapy remains the mainstay in the ventilation strategy for ALI/ARDS treatment, as the increased arterial oxygenation is desired to uphold lung viability. Hyperbaric oxygen therapy (HBOT) is a therapeutic administration of 100% oxygen intermittently under the elevated atmospheric pressure, by which patients are given high concentration and pressure of oxygen inside an enclosed chamber [131]. As an adjuvant treatment, hyperbaric oxygen alleviated a variety of hypoxia-related tissue injuries and enabled the recovery of wound healing by sufficing the oxygen supply to the injured sites [132]. In animal models of endotoxin-induced ALI, HBOT substantially reversed hypoxemia and improved the survival rate, possibly through production of nitric oxide and reduction of oxidative stress [133, 134]. At the cellular level, HBOT increased the bioavailability of intracellular oxygen contents and recovered the membrane integrity to restore mitochondrial respiratory chain, thereby deactivating the caspases and lessening the apoptotic cell death [135, 136]. Despite the concerns that hyperbaric oxygen exposure may heighten the risk of oxygen toxicity, HBOT preserved pulmonary functions in patients with or without pre-existing respiratory diseases, and palliated severe patients with COVID-19 in their disease progression to respiratory failure [137, 138].

In addition to oxygenation and ventilation strategies, pharmacological interventions aim to eliminate edema fluid, relieve pain/discomfort, and treat the underlying cause of lung injury. To date, several drugs have been studied for human ALI/ARDS treatment, including vasodilators, anti-inflammatory drugs, surfactant therapy, diuretics, and antibiotics (Fig. 1) [139]. Table 1 also summarizes the different types of pharmaceutical agents, their mechanisms of action, and the major outcomes of human trials. When considering different pathogeneses of ALI/ARDS with pulmonary or extrapulmonary origins, the effectiveness of the same pharmacotherapy on patients with different ALI subtypes can vary completely or partially. For example, inhaled drugs directly targeting the lung epithelium may be more effective in treating ALI of pulmonary insults, whereas intravenous injection to quench inflammatory responses at the pulmonary capillary endothelium may be more effective in treating ALI of extrapulmonary origin. Nitric oxide inhalation can dilate the pulmonary vasculature and reduce the intrapulmonary blood shunting as patients with extrapulmonary ARDS responded worse than those with pulmonary ARDS [122]. Simultaneously, daily intravenous macrolide injection significantly reduced 30-day mortality in patients with extrapulmonary but not pulmonary ARDS [140]. Similarly, research on pediatric patients found that endothelial activation and inflammation (e.g., IL-6) were frequently linked to indirect rather than direct ARDS causes [141, 142]. This distinct inflammatory pattern was discovered in murine models of both pulmonary and extrapulmonary ALI [143]. Anti-inflammatory therapy is thus expected to have a better or faster effect in patients with indirect ALI.

Notwithstanding, there have been very few studies that assess the differential pharmacological effects between patients with direct and indirect ALI. Furthermore, there is no clinically approved drug that can significantly reduce ALI/ARDS mortality, though some pharmaceutical agents may be more responsive or effective in one subtype than the other [144]. There is still debate in the literature about whether one medication can reduce the mortality rate of hospitalized patients with ALI. The reasons are numerous. Firstly, different administration routes, doses, timing, and duration of the same drug may have distinct effects in treating ARDS of various origins [145]. Secondly, regardless of whether patients have extrapulmonary or pulmonary ALI/ARDS, a variety of harmful causes can coexist, and different stages of ALI may necessitate personalized care and treatment. Third, potential toxicities and side effects of drug agents may exacerbate the already complex heterogeneity of disease development in ALI/ARDS patients.

Hence, the active search for successful ALI/ARDS therapeutics remains critical, necessitating a better understanding of this devastating disease as well as an optimized assessment of clinical trials. To increase the likelihood of a positive outcome in a clinical trial, strategies are suggested to increase sample size, reduce variation (e.g., minimize heterogeneity of enrolled patients), and enhance therapeutic effect (e.g., selecting appropriate ALI phase and implementing multiple interventions) [146].

### Comparative medicine for ALI/ARDS

Extensive research has been conducted in animal models of ALI, but the vast majority of them have resulted in unsuccessful clinical trials. The initially effective therapeutic candidate improved several secondary endpoints, such as ventilator-free days and oxygenation indices, but it failed to statistically improve patient survival. This translational gap is caused by the difficulty with which researchers could simulate human ALI/ARDS in animal studies. Thereby, an urgent question arises regarding how to interpret and minimize interspecies variability between animal models and human patients with ALI.

ALI/ARDS research has used a variety of animal models, including mice [147], sheep [148], and nonhuman primates [149]. When selecting an appropriate ALI model, several critical variables must be optimized, including animal size, lung anatomy, innate immunity, biochemical pathway, and availability of species-specific measures [31]. For instance, large animals such as horses, can provide the necessary amount of blood or BALF sample for investigations, but inflammation measurements from uncommon source (e.g., ovine, equine) can be difficult to obtain [63]. Furthermore, large ruminants (e.g., ovine) have segmented lungs with distinct pulmonary circulation from other mammals, implying a divergent immune response to lung insults. For example, pulmonary intervascular macrophages were found in ruminants and pigs but not in rats or primates/humans, indicating that those animal models are more vulnerable to lung injury when challenged with endotoxins via i.v. injection [150]. Given the high and time-consuming costs of developing such a model, as well as the lack of intensive care experience in large animal research, efforts to develop a large-size animal model of ALI/ARDS are still discouraged.

Oppositely, small animals (e.g., rats, mice, rabbits) have several obvious advantages, including easy breeding, low economic and time expenditure, and relatively high research throughput and data reproducibility, which encourages their widespread use in biomedical translation. Of them, the mouse models of human diseases have been widely used, with a comprehensive understanding of genetics and proteomics in mice, and an abundance of species-specific testing reagents and methodologies. Nonetheless, the body size and mass of small rodents may result in two flawed outcomes that could stymie ALI/ARDS research. On the one hand, the animal sensitivity to dosage range or degree of severity may be the least differentiable when ALI models are built. On the other hand, the obtained BALF and blood samples from mice may be insufficient.

**Fig. 2:**
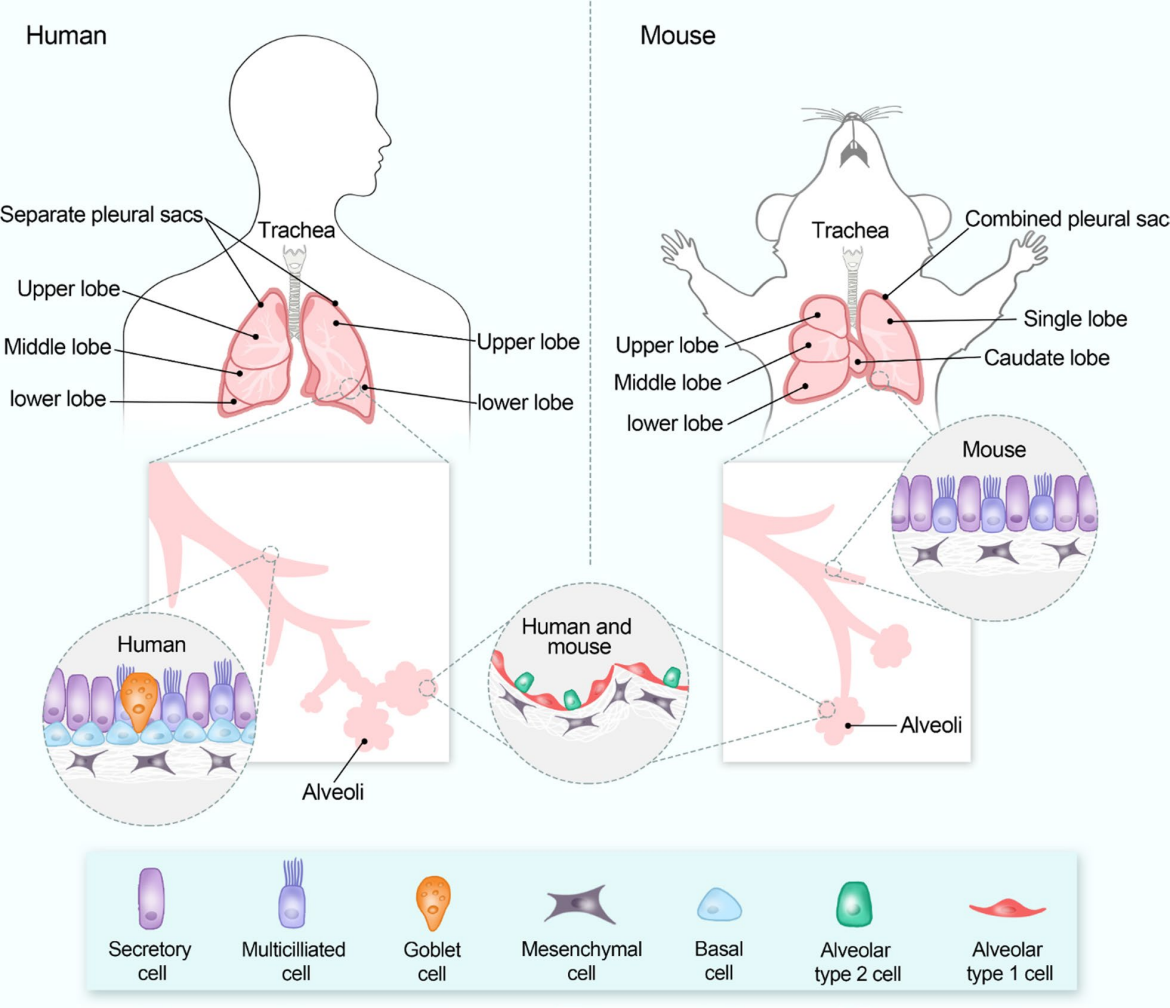
Major differences in anatomical features of the human and mouse lungs. More comparisons of microscopic airway anatomy and biochemical characteristics between humans and mice were reviewed elsewhere [43, 154]

Furthermore, many mouse-human differences must be considered when extrapolating laboratory findings to clinical significance. First, mice are raised in confined facilities where risk factors for human ALI cannot be spontaneously induced. For example, it is impossible to recreate the comorbidity and medical history of ALI patients in mice [151]. Second, the anatomical structures and immune responses between of mouse and human differ significantly, resulting in divergent pathological patterns and pathways (Fig. 2). For example, the formation of hyaline membrane, which was common during the exudative phase of human ALI, was rarely observed in mice [152]. Simultaneously, the immune response to oxidative stimuli in murine and human lung injury varies [153]. Even when using different mouse strains, their susceptibility to the same pathogens or endotoxin challenges can differ. Last but not least, the real-life ICU scenario in which patients with ALI/ARDS were treated with a combination of pharmacological and non-pharmacological interventions is difficult to replicate in mouse models, and endpoint assessment in experimental models does not accurately reflect the disease progression in human ALI/ARDS [154].

All of these elements contribute to the failure of experimental drug candidates in human trials. Because no single animal model can replicate all human ALI traits, using mice to study human ARDS should always be done while accepting and recognizing cross-species variation [153]. The American Thoracic Society has issued official recommendations on the characteristics and experimental measurements of ALI animal models, which include four main categories: histological evidence of tissue injury; alteration of the alveolar-capillary barrier; presence of an inflammatory response; and incidence of physiological dysfunction [34, 155]. Within 24 h of the onset of ALI, at least three out of four events should be determined, although unlisted events cannot be ruled out.

### Nanomedicine for ALI/ARDS

**Fig. 3:**
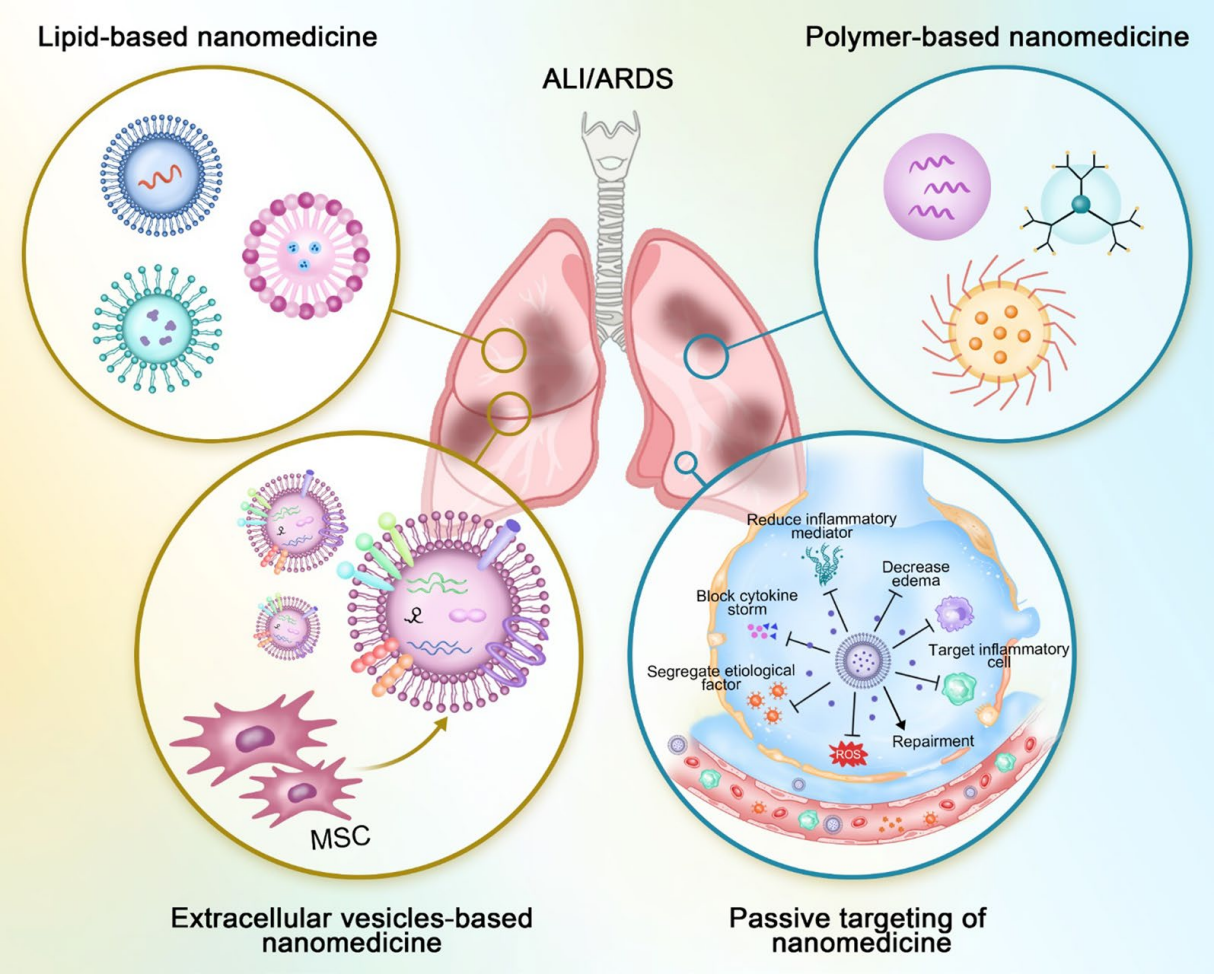
Nanomedicine for ALI/ARDS. Various nanomaterials can be fabricated, including biogenic extracellular vesicles and synthetic lipid (liposome) and polymer nanoparticles, to intervene in the pathophysiological processes of ALI/ARDS for enhanced treatment

In addition to the drawbacks of translational research, the current failure of pharmacotherapy on human ALI/ARDS stems in part from obstructed pharmaceutical delivery to pulmonary sites and the unavailability of drug accumulation in disease lesions; that is, we are still dealing with traditional systemic drug delivery issues. To address this issue, pharmaceutical modifications using nanotechnology open up a new avenue for improving pharmacological outcomes while reducing side effects. The common strategies include the surface decoration to increase circulation time and targeting efficiency, size manipulation to control penetration through biological barriers or accumulation inside treating sites, and multiple functionalities at one hit for theranostic or synergistic purposes, where biodegradable and biogenic materials could benefit even more given the potential clinical translation (Fig. 3).

### Extracellular vesicles-based nanomedicine

Stem cells, particularly mesenchymal stem cells (MSCs), have long been regarded as protective and repairing therapeutics for ALI/ARDS due to their widely accepted safety and efficacy [156]. However, given their potential tumorigenesis, high immunogenicity, and instable hemodynamics, MSC-derived extracellular vesicles (EVs) are emerging and growing substitutes [157]. Most types of cells secrete EVs, which are plasma membrane-bound anuclear particles that carry a variety of biomolecules (e.g., nucleic acids, proteins) for intercellular communication [158]. Nano-scaled EVs outperformed parental cell therapy in ALI/ARDS treatment, possibly due to improved uptake by receptor immune cells and enrichment of anti-inflammatory microRNAs or/and regulatory proteins [157].

In LPS-nebulized mouse models of ALI, MSC-derived EVs via both intravenous or intratracheal administration showed comparable treatment as MSCs by transferring miR-27a-3p to alveolar macrophages where the nuclear factor kappa B subunit 1 (NFKB1) was downregulated to shift macrophage polarization from proinflammatory to anti-inflammatory phenotype, reducing inflammation and alleviating ALI [159]. In ALI mice induced by subcutaneous sulfur mustard injection, bone marrow MSCs-derived small EVs increased protein expression of epithelial barrier proteins such as E-cadherin, claudin-1, and G protein-coupled receptor family C group 5 type A (GPRC5A), and promoted the expression of Bcl-2 and tight junction proteins via the YAP signal pathway, thereby resisting cell apoptosis and repairing alveolar barrier function [160]. In ALI murine models, small EVs from either adipose-derived MSCs [161] or endothelial progenitor cells [162] that secrete a variety of biomolecules (e.g., hormones, microRNAs) via various signaling pathways resulted in similar endothelial restorations. Furthermore, in the lung tissues of intratracheally LPS-instilled mice, where mitochondrial respiration and ATP synthesis were significantly impaired, causing alveolar membrane disruption, MSC-sourced EVs administered intravenously re-established the integrity of the alveolar-capillary barrier through mitochondrial transfer, thereby attenuating lung injury [163].

EVs of various biological origins contain a large body of various bioactive substances, resulting in distinct therapeutic effects in a variety of ALI models. Simultaneously, engineered EVs with altered interior contents or/and surface properties have been used to treat ALI animals. Primary fibroblasts were transduced with β-catenin and the transcription factor gata4, and their derived EVs were supplemented with functional miRNAs with anti-inflammatory and anti-fibrotic effects in ALI mice [164]. Similarly, thrombin-activated mouse platelets were separated, and an anti-inflammatory drug was added to the in vitro platelet incubation to collect platelet-derived EVs in the supernatant, selectively delivering to the pulmonary site and quenching the cytokine storm in ALI mice [165]. Recently, the receptor-binding domain (RBD) of viral spike protein in SARS-CoV-2 was genetically incorporated onto the external surface of 293 T cell-derived EVs, and such RBD-tagged EVs could be loaded with functional miRNAs and specifically delivered into mice expressing hACE2 [166].

In the meantime, cell membrane-enclosed nanocarriers represent a new biomimetic platform with advantages in pharmaceutical delivery to treat a variety of diseases, including ALI/ARDS. Firstly, particles camouflaged by a single type of cell membrane take advantage of its unique surface properties, resulting in increased bloodstream circulation, improved targeting specificity, and decreased immune resistance. For example, nanosized vesicles coated with platelet membrane accumulated in the lungs when inhaled, and the delivered drugs reduced proinflammatory cytokine levels in ALI mice [167]. Second, hybrid ligands derived from various cell membranes could provide a versatile combination of active multiple targeting. Nanoparticles coated with fused membranes from macrophages and 4T1 breast cancer cells demonstrated high accumulation and uptake in metastatic tumor nodules of mouse lungs in vivo*,* with a marked significant effect [168]. Furthermore, nanoparticles made by fusing EVs secreted from ACE2-rich 293 T cells and human monocytes were aerosolized to effectively lure SARS-CoV-2 binding and neutralize inflammations in LPS-induced ALI mice [169]. Nonetheless, these cell membrane coating strategies face several challenges in biomedical applications, including a limited selection of cell types and the possibility of immunogenicity/carcinogenesis due to certain cell origins.

### Lipid-based nanomedicine

Lipid-based nanoparticles (LNPs) are the most common non-viral vectors that balance biosafety and delivery efficacy, and their widespread use in preclinical and clinical settings has endowed them with well-studied biological properties such as pharmacokinetics and toxicity profiles [170]. For example, a PEGylated phospholipid, namely 1,2-distearoyl-sn-glycero-3-phosphatidylethanolamine-N-[methoxy(polyethylene glycol)-2000] (DSPE-PEG_2k_) self-assembled with human glucagon-like peptide-1 (GLP-1) to form micelles (hydrodynamic size =  ~ 15 nm) in aqueous solutions, which was subcutaneously injected into ALI mice after exposure to aerosolized LPS, showing dose-dependent reversal in lung hyper-inflammatory responses [171].

LNPs are commonly formulated with ionizable lipids as the main frame, cholesterol as the stuffing materials, helper lipids to improve cellular uptake, and polyethylene glycol (PEG)-lipid to reduce protein opsonization and immune clearance. LNPs' precise composition can be tailored, determining their physicochemical properties (e.g., surface charge, particle size) and physiochemical properties (e.g., circulation half-time, disease lesion targeting) [172]. An optimized formulation of LNPs containing various phospholipids, PEG-lipids, and cholesterols successfully encapsulated and delivered messenger RNA (mRNA) of haemagglutinin antibody to the mouse lungs infected by a lethal subtype of influenza A virus via nebulization, significantly improving survival [172].

Among LNPs, liposomes are unique in that they have a vesicular structure of bilayer phospholipids with a hollow sphere in the core where hydrophobic or hydrophilic cargoes can be inserted into the lipid layer or aqueous compartment. In mice challenged by intratracheally administered LPS, intravascular injection of PEG-liposomes loaded with hydrophobic superoxide dismutase/catalase mimetic and functionalized with antibodies to platelet-endothelial cell adhesion molecule on surface, demonstrated particulate accumulation in lungs, anti-inflammatory effects of antioxidant interventions, and potentials to further load hydrophilic drugs to complement lung therapy [173]. Recently, liposomes encapsulating both hydrophobic methylprednisolone (anti-inflammation steroid) and hydrophilic N-acetyl cysteine (mucolytic agent) significantly reduced pro-inflammation cytokines and mucus secretions in the LPS-induced mouse lungs, where intravenous and endotracheal administration caused high particle accumulation in lung endothelium and epithelium, respectively [174].

Due to their non-immunogenicity, exceptional biocompatibility, and loading capacity, LNPs have become the most successful mRNA delivery vehicle entering the clinic. Amidst COVID-19 pandemic, two mRNA vaccines, the BNT162b2 (Pfizer-BioNTech) and mRNA-1273 (Moderna), have been granted Emergency Use Authorization by the United States Food and Drug Administration (FDA). LNP-encapsulated mRNA-based vaccines encoding SARS-CoV-2 spike proteins demonstrated significant efficacy in preventing viral infection, reducing the severity of COVID-19 illness, and lowering COVID-related mortality [175, 176].

### Polymer-based nanomedicine

Natural or synthetic polymers are a class of materials that contain repeating chemical units. A variety of differently structured polymers, have been used as pharmaceutical nanocarriers, such as linear and branched polymers, block copolymers, and dendrimers. In pulmonary delivery to treat ALI/ARDS, polymeric materials are used as drug carriers to target or respond to inflammatory microenvironment such as hypoxic and acidic surroundings, excessive enzymes, migrated immune cells, and elevated proinflammatory cytokines [177].

In a recent study, an amphiphilic copolymer poly(β-amino esters)-polyethene glycol (PAE-PEG) was used to entrap the hydrophobic (2-[(aminocarbonyl)-amino]-5-(4-fluorophenyl)-3-thiophenecarboxamide (TPCA-1), and the biotinylated PEG were further conjugated to avidin-tagged intercellular adhesion molecule-1 (ICAM-1) antibody for lung endothelium targeting in LPS-nebulized ALI mice, where pH-sensitive PAE degraded upon acidic environment in inflamed lungs and thus released TPCA-1 to quench the cytokine production [178]. Similarly, anti-inflammatory glucocorticoid dexamethasone was loaded into a mixture of poly(1,4-phenyleneacetonedimethylene thioketal) and polythioketal urethane to form nano-scaled particles, and the polymers' abundant thioketal bonds broke down when activated by ROS in LPS-impaired lungs after i.v. administration [179]. As a result, the dexamethasone released on site effectively cleared alveolar edema and thwarted inflammatory cell infiltration, alleviating ALI.

In addition to pH and ROS, such stimuli-responsive polymer nanoparticles for ALI treatment could be designed and prepared in response to an inflamed lung-associated pathological milieu, such as redox changes, protein overexpression, and neutrophil migration. However, it has been discovered that polymers, particularly cationic polymers, may cause ALI per se by deactivating critical proteins in the airways [180]. Therefore, further development of polymeric nanomaterials for pulmonary delivery necessitates extensive toxicity testing and biosafety evaluation.

## Conclusion and perspective

ALI is a complex illness with numerous causes, whereas ARDS is a diffusive and severe form of ALI that claims millions of lives worldwide each year. During the COVID-19 pandemic, ALI/ARDS progressed from SARS-CoV-2 induced pneumonia has become a significant cause of COVID-related death. Only supportive care is currently the mainstay of ALI/ARDS treatment, and no clinically approved pharmacotherapy is available, although many drug candidates demonstrated significant efficacy in preclinical tests and early phases of clinical trials. This real-world quandary stems primarily from the heterogeneous nature of ALI disease, as well as the pathophysiological difference between human ALI and animal models. Many unresolved issues remain in ALI research, such as specific signaling pathways, environmental and genetic contributions, and the combination strategy of ventilation and medication. On the plus side, nanosized delivery systems made of advanced biological or synthetic materials hold great promises for precise targeting and controlled release of anti-inflammatory drugs in injured lungs. However, whether those delivering platforms caused topical/systemic toxicity or any long-term negative effects warrants continued research attention and effort.

In comparison to the heterogeneity and complexity of ALI therapy, the current ALI diagnosis criteria are relatively simple. Novel visualization techniques to diagnose ALI with a link to the specific ALI cause are highly desired to help understand the pathogenetic mechanism and differentiate the subtypes of ALI/ARDS. This new imaging modality should be capable of monitoring in vivo lung pathophysiology in real-time. Such research should be implemented in clinical trials to correlate therapeutic effects with patient clinical manifestations. Furthermore, biopsy and autopsy of tissue samples from ALI patients to refine the disease phenotype would be extremely beneficial. Simultaneously, because clinical trials are always labor-intensive, time-consuming, and expensive, emerging big data technology such as machine learning may be an essential complement to improve rational design for ALI/ARDS treatment.

## Data Availability

Not applicable.
